# High Intensity Functional Training in Hybrid Competitions: A Scoping Review of Performance Models and Physiological Adaptations

**DOI:** 10.3390/jfmk10040365

**Published:** 2025-09-24

**Authors:** Paula Villarroel López, Daniel Juárez Santos-García

**Affiliations:** Sports Training Laboratory, Faculty of Sport Sciences, University of Castilla-La Mancha, 45071 Toledo, Spain; paula.villarroel@alu.uclm.es

**Keywords:** CrossFit, concurrent training, athletic performance, neuromuscular adaptations, fatigue

## Abstract

High-Intensity Functional Training (HIFT) is a training method that has garnered increasing attention due to the rise in hybrid competitions such as CrossFit or Hyrox, a race format combining strength and endurance tasks in a fixed structure. Therefore, an integrative approach is needed to help us understand which physiological capacities this training method enhances. **Objectives:** This scoping review aimed to map the current scientific literature related to HIFT, with a particular focus on physiological and psychobiological determinants of performance in hybrid competition contexts. **Methods:** Following the methodological framework of Arksey and O’Malley and the PRISMA-ScR guidelines, a systematic search was conducted in Web of Science, Scopus, and PubMed. Thirty-nine studies published between 2015 and 2025 were included. **Results:** HIFT was found to improve key physical attributes such as aerobic capacity, muscular strength, anaerobic power, and fatigue tolerance. Increases in VO_2_max ranging from 8% to 15% and strength gains of 10% to 20% in major lifts were commonly reported. Improvements in local muscular endurance, power output, and recovery capacity were also observed. The physiological benefits appeared more pronounced in trained individuals, especially those with greater resistance training volume. In addition, psychobiological responses, including perceived exertion, cognitive control, and motivation, were explored in several studies, with more experienced athletes showing higher fatigue tolerance and better performance consistency under stress. **Conclusions:** HIFT enhances essential physical attributes applicable to hybrid events. The findings support the use of HIFT as a foundational method for training athletes involved in demanding multi-domain fitness settings, without attributing these benefits specifically to any single competitive event.

## 1. Introduction

Performance in hybrid sport disciplines, defined as competitive formats that combine cyclical endurance elements (e.g., running, rowing) with functional strength tasks (e.g., weightlifting, carries, callisthenics) within a fixed or timed structure, depends on the integration of strength and endurance capacities, which raises the challenge of concurrent training, defined as the combination of strength and endurance exercises in the same session or within a periodised programme [1,2]. Theoretical frameworks such as the interference effect and the theory of adaptation pathways are essential to understanding the physiological complexity of concurrent training, especially in hybrid formats. The interference effect refers to the idea that combining strength and endurance training, particularly when done in the same session or with insufficient recovery, can limit the full development of one or both capacities. For example, intense endurance training may reduce the gains typically expected from strength training. The theory of adaptation pathways complements this by proposing that the body adapts differently depending on the type, order, and intensity of the training stimulus. When multiple demands are placed on the body at once, the way they are sequenced can influence which adaptations dominate. In hybrid competitions, where strength and endurance are constantly mixed with little rest, understanding these principles helps coaches and athletes structure training to improve both capacities without compromising performance [3,4].

Within the framework of high-intensity training, HIFT (high-intensity functional training) has gained popularity as a method that integrates functional movements with high loads and minimal rests, with the aim of improving general and functional fitness [5].

This approach incorporates elements of weightlifting, cardiovascular conditioning and gymnastic exercises, optimising physical domains such as strength, coordination, speed and agility [6]. Due to the rise in these HIFT methods, hybrid competitions are becoming increasingly popular in the world of competitive sports. One of the best-known sports in this field is CrossFit. This sport incorporates routines named “WOD” (Work Of the Day), which combine varied functional movements performed at high intensity [7].

Another hybrid competition that is growing in popularity is HYROX, which integrates running with structured functional exercises. HYROX, for instance, is a well-known competitive event that integrates running intervals with functional exercises in a fixed sequence. What differentiates HYROX from CrossFit is its standardised format and the specific combination of prolonged endurance (8 × 1 km runs) with repeated high-intensity tasks performed with minimal recovery time between them (SkiErg, Sled Push/Pull, Burpee Broad Jump, RowErg, Farmers Carry, Sandbag Lunges and Wall Balls). Recent studies have begun to analyse the acute physiological responses and performance factors associated with HYROX identifying its main physical demands and suggests the need for specific preparation strategies [8].

Despite widespread use, few reviews systematically map physiological and psychobiological determinants of performance in HIFT or hybrid formats. Given the increasing popularity of HIFT-based competitions, understanding performance indicators is necessary to optimise science-based programming and training.

Considering all of this, this scoping review aims to explore the literature on physiological adaptations, performance predictors, and psychobiological responses associated with HIFT. By focusing on both general HIFT practice and its implementation in structured competitions, the review seeks to support practitioners in optimising training protocols.

## 2. Materials and Methods

### 2.1. Study Design

The SALSA structure [9] was used for the development of this review. This framework uses four critical phases (Search, AppraisaL, Synthesis and Analysis) to both guide and evaluate a scoping review. This scoping review was conducted in accordance with the PRISMA-ScR (Preferred Reporting Items for Systematic Reviews and Meta-Analyses extension for Scoping Reviews) guidelines [10]. The review protocol was not registered in any public database.

We followed the 5 stages of the methodological framework for scoping reviews Arksey and O’Malley [11] and the recommendations and additional proposals of Codina [12]: (1) identification of the research question (s); (2) search for relevant studies; (3) selection of studies; (4) data extraction and analysis; and (5) collection, synthesis and presentation of results.

The choice of a scoping review is motivated by the need to map an emerging field (HIFT or hybrid formats), where methodological diversity, heterogeneity of studies and the lack of a consolidated empirical basis make the use of traditional systematic reviews or meta-analyses unsuitable.

### 2.2. Eligibility Criteria

The following inclusion criteria were established:

1. Studies published from 2015 onwards.
2. Languages: English or Spanish.
3. Participants aged 16 and over.

Thematic focus on High Intensity Functional Training (HIFT), CrossFit, concurrent training or HYROX-type hybrid competitions.

Studies related to clinical populations (e.g., chronic disease, cancer, Parkinson’s), pregnancy, injury prevention, nutrition or interventions not directly related to performance or physical training were excluded.

### 2.3. Information Sources and Search Strategy

The literature search was conducted between December 2024 and May 2025 in three databases: Web of Science, Scopus, and PubMed. Search terms were combined using Boolean operators and adapted for each database. The complete search strategy included the following string: (“concurrent training” OR “Crosstraining” OR “high intensity functional training”) AND (“CrossFit” OR “HYROX” OR “hybrid competitions”). Filters were applied to include only peer-reviewed journal articles published in English or Spanish between 2015 and 2025. A total of 331 records were retrieved: 122 from Web of Science, 117 from Scopus, and 92 from PubMed. After removing 192 duplicates, 139 articles were screened based on title and abstract. Studies were included if they (1) investigated high-intensity functional training (HIFT) protocols or hybrid training modalities (e.g., CrossFit, HYROX), (2) reported physiological or psychobiological outcomes, and (3) involved healthy adult populations. Exclusion criteria were: (a) studies not published in English or Spanish, (b) lack of access to full text, (c) non-peer-reviewed sources, and (d) studies that did not address physiological or performance-related variables. Following screening and full-text assessment, 39 studies met the inclusion criteria and were analysed in the review.

### 2.4. Selecting Sources of Evidence

The results of the searches were exported to EndNote^TM^20 for automatic removal of duplicates. Titles and abstracts were then independently assessed by two reviewers. Those studies that met the criteria were read in full text to confirm inclusion. Discrepancies were resolved by consensus or, if necessary, by consulting a third reviewer.

### 2.5. Data Mining

The data were organised and recorded in an Excel spreadsheet. For each study included, the following variables were extracted: author/year, study design, sample characteristics, objectives, methodology, main findings and conclusions. 

### 2.6. Quality Assessment

The quality of the included studies was assessed using the AXIS (Appraisal Tool for Cross-Sectional Studies) tool, developed specifically to evaluate cross-sectional studies.

This tool allows common methodological weaknesses to be identified through 20 items covering key aspects such as: clarity of objectives, appropriateness of design, justification of sample size, sample selection process, validity of measures used, and transparency in the presentation of results [13].

For interpretive purposes, the studies were classified into three levels according to the percentage of items met: high quality (≥75%), moderate quality (50–74%), and low quality (<50%). This categorization was not used as an exclusion criterion, but it did allow for contextualising the findings and qualifying the degree of confidence in the results.

## 3. Results

**Figure 1:**
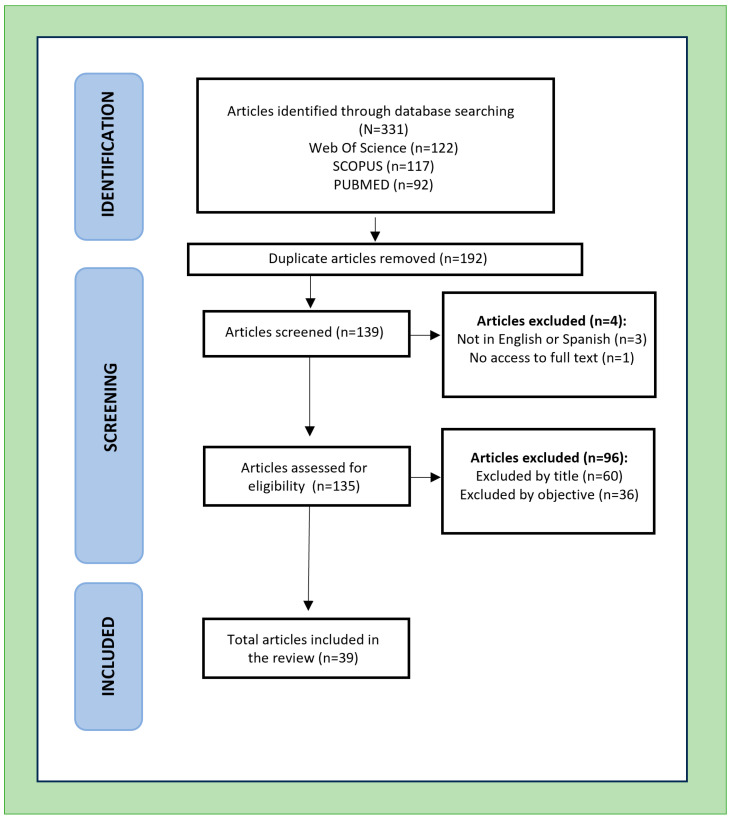
Flowchart.

The PRISMA flow chart summarising the study selection process is presented in Figure 1.

After applying the inclusion and exclusion criteria, 39 articles were selected for analysis (Table 1):

**Table 1:** Summary of study characteristics.

Author and Year.	Sample	Objective	Methodology	Results and Conclusions
Wang, Soh [14]	9 high quality research, 911 healthy individuals of various ages and training levels, including high school students, firefighter recruits, cadets and athletes from disciplines such as judo and taekwondo.	To assess the effects of high-intensity functional training on physical fitness in healthy individuals, analysing multiple components of physical performance.	Systematic review and meta-analysis (PRISMA) with search in five databases and quality assessment with TESTEX, ROB 2 and GRADE. Strength, power, speed, speed, endurance, agility and flexibility were analysed.	HIFT significantly improves strength, power, speed, endurance and agility, but does not affect flexibility.
Brandt, Ebel [8]	11 Hyrox© recreational athletes (27% female), with around 18 months of experience.	To analyse the acute physiological responses and determinants of performance in a simulated Hyrox competition.	VO_2_max, body composition and strength were assessed; they then performed a full Hyrox simulation with continuous measurement of heart rate, lactate and RPE.	The test had a high cardiovascular demand (80% in the very high HR zone). VO_2_max, low fat % and resistance training volume correlated with better performance. Wall balls were the most demanding exercise. Hyrox is a predominantly aerobic modality with high functional demands, which makes it suitable both for health promotion and for people who require a high level of fitness in their work, such as security, emergency or armed forces personnel.
Santos, Morais [15]	21 healthy, trained men. 2 sessions of 20 min on separate days: one HIFT and one HICT.	To compare physiological and psychobiological responses during and after a HIFT session with a HICT session.	Heart rate, blood lactate, anxiety, perceived exertion and discomfort were measured. HICT intensity was adjusted to match the mean HIFT heart rate.	Maximum heart rate, blood lactate concentration and perceived exertion were significantly higher in HIFT than in HICT (*p* < 0.05).
Ponce-García, García-Romero [16]	50 CrossFit athletes (25 men and 25 women) with an average age of 33.3 years. Men had higher body mass and height than women.	To determine the differences in anaerobic performance between sexes in CrossFit athletes, evaluating absolute and relative values in different maximal effort tests.	Cross-sectional study that assessed body composition and anaerobic performance using three maximal exercise tests: Wingate, Repeated Jumping and Anaerobic Squat Test.	Men showed higher peak and mean power in all tests, although the differences decreased when adjusting for lean and muscle mass. Mean power remained higher in men, except in the adjusted squat. Females showed greater fatigue in repeated jumps, with no differences in other tests.
Tibana, Dominski [17]	14 male volunteers, with an average age of 30.3 ± 5.8 years. At least two years of CrossFit experience and attended more than four training sessions per week.	To examine the assessment between anthropometric measures, cardiorespiratory fitness, muscular power, local muscular endurance and Total Athleticism Score (TSA) with performance in the CrossFit Open 2021.	Participants were tested in different tests separated by weeks, including fat percentage, maximal oxygen consumption, muscle power (power clean) and local muscular endurance (Tibana Test).	A high correlation (*r* = 0.91; *p* < 0.01) was found between TSA and CrossFit Open 2021 z-scores, with the best and worst scores overlapping in both.
Martinho, Rebelo [18]	68 investigations analysed adults with CrossFit experience, considering diverse demographic characteristics and levels of competition, providing a comprehensive view of the physical demands and physiological responses in these practitioners.	To synthesise the current evidence on physical demands and physiological responses to CrossFit, identify gaps in the literature and examine correlations between different physiological variables and CrossFit performance.	A review was conducted following PRISMA 2020, with searches in PubMed, Scopus and Web of Science. Studies on physical and physiological parameters in adult CrossFit^®^ practitioners were included, and a meta-correlation was performed to analyse predictors of performance.	Studies show that physical and physiological markers vary according to the type of CrossFit training, indicating specific demands on the body. Between 48 and 72 h are required for full recovery after intense sessions. Benefits in muscle hypertrophy and strength are similar to those of concurrent training.
D’Hulst, Hodzic [19]	The sample consisted of 60 high performance CrossFit^®^ athletes, men and women from the top 5% of the CrossFit Open, including 7 semi-finalists and 3 finalists from the CrossFit Games.	Establish detailed physiological profiles of highly trained CrossFit^®^ athletes and determine which physiological markers correlate best with CrossFit Open performance.	Peak leg and trunk strength was measured with dynamometers, jump height and traction force with a force platform. Peak VO_2_ was assessed in a cardiopulmonary test and critical power and W’ in a 3 min cycloergometer test.	Men had a peak VO_2_ of 4.64 L∙min^−1^ and women 3.21 L∙min^−1^. Critical power was 314.5 W in men and 221.3 W in women, and traction force was 3158 N in men and 2035 N in women. Regression showed that anthropometric variables were associated with CrossFit performance, but only in women was the relationship with peak VO_2_ significant.
Wang, Soh [20]	13 high quality investigations, with 478 athletes aged 10 to 24.5 years testing various sport disciplines, including judo, taekwondo, wrestling and football.	To assess the impact of HIFT on the general fitness and sport-specific performance of athletes.	A systematic review and meta-analysis was performed following PRISMA, searching five databases. The methodological quality of the studies was assessed using the PEDro scale. Variables such as strength, power, speed, endurance, agility and flexibility were analysed.	The meta-analysis showed that HIFT improves strength, power, speed, endurance and agility, with significant effects in these areas. However, it has a minor impact on flexibility, suggesting that specific exercises are needed to improve flexibility.
Sant’Ana, Evmenenko [21]	33 participants (22 men and 11 women) with an average age of 34.9 years, weight of 72.3 kg, height of 1.72 m and a BMI of 24.4 kg/m^2^. They were experienced in HIFT and performed 60–90 min sessions.	To assess autonomic responses and internal load through heart rate variability (HRV) during a HIFT session.	Heart rate variability (HRV) was analysed at three points in time: specific warm-up, exercise phase (50 min) and recovery phase (10 min). A Polar H10 heart rate monitor and the Elite HRV application were used, transferring the data to the Kubios HRV Standard software version 3.3.1. for processing.	HIFT can alter HRV, affecting autonomic behaviour, and that this type of training can provide significant levels of load, affecting physiological responses and, consequently, the individual’s functional efficiency.
Pearson, Olenick [22]	13 CrossFit^®^ practitioners (24.5 years, 75.2 kg) with more than one year of experience, and 8 sedentary people (24.3 years, 77.3 kg) who did less than 2 h of structured exercise per week.	To assess aerobic capacity, metabolic response during high-intensity exercise, resting mitochondrial oxidative capacity and resting vascular function in adult CrossFit^®^ exercisers and a sedentary group.	Maximal aerobic capacity (VO_2_ peak), substrate oxidation during high-intensity exercise, resting mitochondrial oxidative capacity and resting vascular function were measured. Oxidation was assessed with indirect calorimetry, mitochondrial capacity with NIRS, and vascular function with flow-mediated dilation (FMD) in the brachial artery.	Chronic participation in high-intensity functional training programmes, such as CrossFit, can improve aerobic capacity, substrate oxidation during exercise, resting mitochondrial oxidative capacity and resting vascular function.
Moscatelli, Messina [23]	No particular sample is specified, as multiple research studies with participants of different characteristics were reviewed.	To provide an overview of heart rate and its implications on aerobic and anaerobic parameters. The aim is to understand how intensity modulation in CrossFit can benefit these physiological aspects.	Narrative review of existing studies on heart rate, focusing on its impact on aerobic and anaerobic capacity. The physiological effects of heart rate and how variation in training intensity can influence these parameters were discussed.	CrossFit is a training modality that, by integrating cardiovascular and resistance exercises, offers both aerobic and anaerobic benefits. The variability in the intensity of the workouts allows CrossFit to be adapted to the specific needs and goals of each individual.
Meier, Sietmann [24]	27 non-professional CrossFit^®^ athletes (18 men and 9 women) with an average age of 30.9 years and 16.1 months of experience, training approximately 2.9 h per week.	To assess how physiological parameters measured by HR vary during four one-hour CrossFit sessions in non-professional athletes and what factors influence these variations.	The one-hour sessions were divided into warm-up (WU), skill and strength development (A), and high-intensity training (B). Heart rate (HR) was measured during each part to assess the intensity and internal load of the training.	HR was significantly higher in part B of the workout compared to parts A and WU. The one-hour CrossFit^®^ sessions combine anaerobic and aerobic intensities, allowing beginners and experienced athletes to train with similar cardiovascular responses and intensity levels.
Meier, Schlie [25]	21 research studies that looked at CrossFit^®^ athletes of different experience levels and physical characteristics. Healthy adult participants.	To identify and summarise predictors of performance in CrossFit^®^, to provide evidence-based recommendations for improving performance in this sport.	Systematic review following PRISMA, with searches in PubMed, SPORTDiscus, Scopus and Web of Science. Studies on physiological, anthropometric and competitive experience parameters related to CrossFit performance were included.	Body composition, especially lean mass and fat percentage, influences CrossFit^®^ performance. Competition experience and total and trunk strength correlated with better results, but no single physiological parameter was identified as a predictor of performance.
McDougle, Mangine [26]	60 articles investigating 35 unique HIFT workouts. Participants ranged in age, gender and fitness level, to include sedentary individuals to trained athletes.	Map and summarise the existing literature on acute physiological responses to HIFT, providing an overview of how this type of training affects the body immediately after it is performed.	Scoping review according to Arksey and O’Malley, searching three databases to identify articles on HIFT. Variables such as immediate cardiovascular, metabolic, hormonal and immunological responses to training were studied.	HIFT significantly increases heart rate, blood pressure, lactate and oxygen consumption, indicating high cardiovascular and metabolic demand. It also elevates adrenaline, cortisol and affects the immune system, suggesting modulation of the immune system.
De Brito, Fernandes [27]	The study included 32 HIFT practitioners, divided into three performance groups: Elite (7 participants, 28.9 years, 50 months of practice, 13.5 h/week), Advanced (10 participants, 33.4 years, 27.6 months of practice, 8.6 h/week) and Beginner (15 participants, 30.6 years, 22.9 months of practice, 4.7 h/week).	To analyse the acute effect of a HIFT session, specifically the WOD “Fran”, on the cognitive functions and physiological parameters of the practitioners, considering their level of performance.	Physiological parameters such as heart rate, blood lactate concentration and systolic and diastolic blood pressure, as well as cognitive functions were assessed using the five-digit test, before and after the performance of the WOD “Fran”. The results were compared between the different performance levels.	The Elite group completed the WOD in less time than the Advanced and Beginner groups. All groups showed significant improvements in executive functions, with differences according to performance level. Increases in post-exercise heart rate and lactate were also recorded, varying by competitive level.
Tibana, de Sousa Neto [28]	Eight healthy, functional fitness-trained men, with an average age of 28.1 ± 5.4 years, a body weight of 77.2 ± 4.4 kg and a VO_2_max of 52.6 ± 4.6 mL∙kg^−1^∙min^−1^	To investigate the temporal effects of a functional fitness training session performed at different intensities on metabolic, hormonal and BDNF responses in trained men.	In a randomised, crossover design, participants performed three sessions: maximal intensity training, self-regulated intensity and non-exercise control. Serum lactate, CK, testosterone, cortisol and BDNF were assessed at five time points: pre-exercise, post-0 h, 1 h, 2 h and 24 h post-exercise.	CK concentrations increased 24 h after both workouts, indicating muscle damage. There was a decrease in testosterone after the all-out session, suggesting further hormonal suppression. Cortisol increased after both sessions, reflecting stress. Only the all-out session significantly elevated BDNF, suggesting an impact on neuroplasticity.
Neto, de Sousa [29]	Eight CrossFit trained men (mean age: 28.4 ± 6.4 years; 1RM in back squat: 139.1 ± 26.0 kg).	To evaluate physiological and perceptual recovery after performing the CrossFit^®^ benchmark “Karen”, which consists of 150 repetitions of Wall Balls with a 9 kg ball, aiming at a target at a height of 3 m.	CMJ height, CK concentration and PRS scale (general, lower and upper extremities) were measured at the following time points: pre-exercise, immediate post-exercise (0 h), 24 h, 48 h and 72 h after exercise.	At 24 h post-exercise, CK concentration increased significantly (338.4 U/L vs. 143.3 U/L; *p* = 0.040). At 48 and 72 h, CK levels returned to baseline values. Perceived recovery scores were significantly lower 24 h after exercise.
Martinez-Gomez, Valenzuela [30]	15 recreational male CrossFit^®^ practitioners (average age: 29 ± 8 years).	To determine whether voluntary exercise or NMES could improve recovery after a HIFT session compared to total rest.	Crossover design, participants performed HIFT and were then assigned to one of three recovery conditions. Perceived exertion, muscle soreness, heart rate, lactate, muscle oxygen saturation and jumping ability were assessed up to 24 h later.	A significant interaction (*p* = 0.035) will be observed, with a trend towards a lower RPE in the NMES condition compared to total rest immediately after the 15 min recovery. No significant effects were found for the other indicators evaluated (all *p* > 0.05).
Luis Mate-Munoz, Budurin [31]	18 strength-trained men, with an average age of 24.22 ± 2.73 years, weight of 76.43 ± 8.22 kg, height of 176.06 ± 4.49 cm, body mass index of 24.55 ± 2.21 kg/m^2^ and a body fat percentage of 13.62% ± 3.27%.	To analyse muscle fatigue and metabolic stress at 15 min of recovery after two independent sessions of Functional Fitness Training (FFT): one session of strength training (FFTstrength) and one session of endurance training (FFTendurance).	Crossover design, participants performed two training protocols (FFT Strength and FFTendurance) with one week of rest in between. Neuromuscular fatigue, metabolic stress, mean heart rate and perceived exertion were measured at different times after training.	Speed in the Squat Test recovered after 15 min rest, while in the Military Press Test it remained 7% lower in the FFTstrength session. Both sessions increased blood lactate to over 12 mmol·L^−1^, indicating high metabolic stress.
Kapsis, Tsoukos [32]	31 young, healthy individuals, randomly assigned to three groups: Moderate load group (ML): 70% of one-repetition maximum (1-RM). Low load group (LL): 30% of 1-RM. Control group (CON): No training intervention.	To assess the effects of two different resistance loads during HIFT on body composition and maximal strength in essentially active men and women.	Each group performed HIFT three times per week for 12 weeks with similar volume. Body composition (fat and lean mass) and maximal strength (1-RM) were measured before and after training.	Both experimental groups significantly reduced body fat after 6 weeks, with the LL group showing a greater decrease. The ML group increased LBM after 6 weeks, and both groups increased LBM at the end of 12 weeks. Both groups also increased maximal strength in several exercises, while the CON group showed no significant changes.
Adami, Rocchi [33]	The study included 30 young men (18–38 years) divided into three groups: 10 HIFT athletes, 10 endurance athletes (END) and 10 weightlifters (POW).	To compare the physiological profile of three age-matched groups of endurance, high-intensity functional training and weightlifting athletes.	Anthropometric characteristics, VO_2_ peak, grip strength, isometric and isokinetic lower limb strength, vertical jump and anaerobic power were assessed by shuttle run test.	NDT and HIFT athletes had higher VO_2_ peak values than POW athletes, with no differences between NDT and HIFT. POW and HIFT athletes showed greater grip strength and jump height than NDT athletes, while HIFT athletes excelled in lower limb isokinetic tests. There were no differences in anaerobic power.
Adami, Rocchi [34]	The study included 20 young men (18–38 years), divided into two groups: Competitive athletes (CMP), with advanced HIFT experience, and Beginner athletes (BGN), with little or no HIFT experience.	To compare the physiological profile of competitive and novice HIFT athletes, assessing differences in aerobic capacity, strength and anaerobic power.	Anthropometry (weight, height, BMI), aerobic capacity (VO_2_ peak), lactate threshold, maximal grip strength, isometric and isokinetic lower limb strength, and maximal anaerobic power (RAST) were assessed.	Competitive athletes (CMP) had higher VO_2_ peak, grip strength and peak power values compared to beginners (BGN), with significant differences in all tests (*p* < 0.001).
Leitao, Dias [35]	The study included 15 male recreational CrossFit^®^ athletes with at least three years of experience and four weekly sessions. Average characteristics were: age 24.03 years, weight 78.2 kg, height 1.75 m, BMI 25.82 kg/m^2^ and body fat percentage 19.39%.	To analyse the physical and physiological variables of recreational CrossFit^®^ athletes during “FRAN” training and to determine which of these variables best predict performance in such training.	Participants performed maximal strength (1RM) and repetition maximal tests on pull-ups and thrusters, in addition to “FRAN” training (21-15-9 thrusters and pull-ups) and a 2K Row test. Blood lactate, HRmax, average HR and RPE were measured before and after each session.	FRAN performance showed a strong correlation with maximal repetitions of thrusters and pull-ups. Maximal strength in thrusters and time in 2K Row were also significantly correlated with “FRAN”. Lactate concentration, HRmax, HRprom and RPE were higher in 2K Row and thruster max repetitions than in “FRAN”.
Hollerbach, Cosgrove [36]	85 healthy university students were divided into two groups: 36 in traditional weight training (TWT) and 49 in high-intensity functional training (CF). The average age was 22.6 ± 4.1 years in TWT and 21.8 ± 3.2 years in CF.	To compare the adaptations in muscular strength, power and endurance between two types of university physical activity classes: TWT and CF.	Measurements were taken before and after an 8-week programme, including vertical jump, grip strength, push-ups, squats, and body composition analysis (weight and fat percentage).	Both groups showed significant improvements in the number of push-ups and squats performed (*p* < 0.001). No significant changes in body fat percentage were observed in either group.
Fernando, Santos [6]	30 participants distributed in two groups: CrossFit^®^ practitioners: 15 individuals. Crosstraining^®^ practitioners: 15 individuals.	To compare the physical capacities and anthropometric measurements between CrossFit^®^ and Crosstraining practitioners.	Vertical and horizontal jump, pull-ups, box jumps, push-ups and burpees were assessed to measure power, strength, muscular endurance and aerobic/anaerobic capacity.	No significant differences in physical capacities and anthropometric measurements were found between CrossFit^®^ and Crosstraining practitioners. Both training methods may have similar effects on the development of physical capacities and on the anthropometric characteristics of the individuals evaluated.
Bustos-Viviescas, Acevedo-Mindiola [37]	20 physically active subjects (10 men and 10 women) with an average age of 24.5 ± 3.5 years. Participants performed the CrossFit ‘Karen’ WOD.	To assess the relationship between continuous and intermittent maximal aerobic speed and performance in the CrossFit ‘Karen’ WOD in physically active subjects.	VAMc, VAMi and performance in the WOD ‘Karen’ (time to complete 150 Wall Balls) were assessed.	VAMi showed a strong correlation with performance in the ‘Karen’ WOD (*r* = −0.74), explaining 54% of the variance in total time. VAMi is a stronger predictor of performance in this WOD than VAMc.
Ben-Zeev and Okun [38]	Review of previous studies on the effects of HIFT on metabolic, cardiovascular and cognitive health.	To explore the molecular mechanisms that explain the benefits of HIFT on health and physical performance.	Analysis of studies on the effects of HIFT on cardiovascular, metabolic and neurological health, including variables such as heart rate, insulin sensitivity and BDNF release.	HIFT improves muscle hypertrophy, cardiovascular function, insulin sensitivity and fat oxidation. It also stimulates neurogenesis and releases BDNF, benefiting memory and learning.
Adami, Rocchi [39]	Twenty HIFT athletes were analysed, 10 males (mean age: 29 ± 5.3 years) and 10 females (mean age: 30 ± 3.2 years).	To characterise the physiological profile of HIFT athletes, analysing their performance in different physical capacities, including strength, endurance, power and body composition.	Cross-sectional study that evaluated aerobic and anaerobic capacity, maximal strength, muscular endurance and body composition in athletes of different training levels, measuring VO_2_max, strength in compound exercises, power, execution speed and fat percentage.	HIFT athletes show high muscular strength and endurance, with performance correlated with anaerobic power. Competitive athletes have better VO_2_max and maximal strength values than recreational athletes.
Zeitz, Cook [40]	24 CrossFit^®^ athletes (12 men and 12 women), with an average age of 28.4 ± 4.7 years, all with at least 2 years of CrossFit^®^ experience and participation in at least one official event.	To examine the relationship between CrossFit^®^ test performance and physiological measures such as aerobic capacity, anaerobic power and body composition, to assess their influence on performance in typical CrossFit^®^ workouts.	VO_2_max, anaerobic power (Wingate test), maximal strength (1RM) and body composition (DEXA) were assessed. CrossFit performance tests included the “Fran” benchmark, burpees test and an Olympic lifting WOD, measuring times, repetitions and total load lifted.	Negative correlation between VO_2_max and time in “Fran” (*r* = −0.72), indicating that athletes with higher aerobic capacity completed training faster. Anaerobic power predicted Olympic lifting performance (*r* = 0.65), and lean muscle mass was positively associated with the load lifted (*r* = 0.78). No significant relationships were found between body fat percentage and performance in endurance or high-intensity WODs.
Schlegel [2]	Twenty-five previous CrossFit^®^ studies were analysed, involving participants ranging from beginners to elite athletes, with sample sizes between 10 and 50 people per study.	To analyse how the combination of strength and endurance exercises in CrossFit^®^ WODs affects physiological adaptations and performance, seeking to identify optimal strategies to maximise strength and endurance without interfering with performance.	Systematic review compiling studies on CrossFit^®^ and HIFT, assessing physiological and athletic performance, focusing on VO_2_max, anaerobic power, maximal strength, muscular endurance and hormonal markers, to analyse the impact of concurrent training on adaptation and recovery.	Concurrent training in CrossFit improves VO_2_max without interfering with strength. Strategies such as undulating periodisation and strength sessions before aerobic work minimise negative effects. Although it does not generate hypertrophy like traditional weightlifting, it improves functional strength. Physiological and hormonal stress is high, requiring proper rest and recovery planning to avoid affecting recovery and performance.
Mangine, Tankersley [41]	16 CrossFit athletes with more than two years of experience. The participants had an average age of 30.7 ± 6.9 years, a height of 171 ± 12 cm and a weight of 78.0 ± 16.2 kg.	To determine the influence of experience and physiological fitness on performance during the 2018 CrossFit Open events.	Several physiological and experiential variables of the athletes were assessed, including: resting energy expenditure, hormone concentrations, body composition, muscle morphology, cardiorespiratory fitness and isometric strength. These variables were measured on two separate occasions prior to participation in the 2018 CrossFit Open.	Body composition, especially body fat percentage, was the strongest predictor of performance in almost all tests analysed. Competition experience was also a significant predictor of performance. Other factors such as vastus lateralis cross-sectional area, respiratory compensation threshold and rate of force development were also associated with performance, although to a lesser extent.
Cavedon, Milanese [42]	The study included 53 CrossFit participants divided into two groups according to weekly training volume: HT (more than 6 h/week) and LT (less than 6 h/week), compared to age- and BMI-matched control groups.	To investigate how different amounts of training affect body composition and performance in HIFT participants, specifically CrossFit practitioners.	Body composition (measured by DXA), physical performance in the “Fran” WOD, lactate and blood glucose levels, and weekly training volume were assessed in CrossFit participants.	The HT CrossFit group showed lower adiposity, higher lean mass and completed the Fran faster than the LT group. There was a positive correlation between appendicular lean mass and Fran performance. Lactate and glucose levels increased after training, but returned to normal after 15 min of recovery.
Neto and Kennedy [43]	Review of existing literature on high-intensity functional training and does not involve a specific sample of participants.	To review current studies on HIFT and assess how this type of training can be used to improve sport-specific performance.	The authors analysed previous studies on HIFT, focusing on fitness components (aerobic capacity, anaerobic capacity, endurance, hypertrophy, strength and power), session design that integrates multiple components, and the benefits of HIFT on sports performance and its efficiency versus other training methods.	HIFT improves multiple components of fitness simultaneously, benefiting athletic performance. It is time efficient, as it develops several physical capacities in a single session. However, more controlled research in athletic populations is needed to evaluate its specific effectiveness on sports performance.
Feito, Giardina [44]	29 advanced CrossFit athletes.	To examine the physiological responses of advanced CrossFit athletes to consecutive Wingate tests with short periods of active recovery and to determine whether these responses can predict performance in a HIFT.	Four consecutive Wingate anaerobic tests (WAnT) and a CrossFit style workout (AMRAP) were performed. Variables measured in the WAnT included VO_2_, RER, HR, PP, AP, FI and TW. During the AMRAP, the total number of repetitions completed was recorded.	Significant differences in anaerobic performance were observed between the WAnT tests, with higher PP, AP and TW values, and lower FI in the first test. VO_2_, RER and HR increased in all four tests. The ability to predict repetitions in AMRAP improved from the first to the third WAnT test, highlighting the importance of maintaining performance and recovering quickly between high intensity efforts for CrossFit performance.
Cosgrove, Crawford [45]	45 participants (51.1% female, mean age = 31.8 ± 13.2 years, weight = 72.5 ± 13.6 kg, height = 1.73 ± 0.10 m). The majority were non-Hispanic Caucasian (87.5%). HIFT experience ranged from 0 to 27 months, with an average of 9.8 (SD = 8.4) months. They attended HIFT classes 4.0 (SD = 1.1) days per week.	To evaluate the effects of six months of HIFT, specifically CrossFit, on various physical capacities.	Participants completed three separate days of assessments in 10 fitness domains before and after participating in the programme for six months. For each sex, a 2 (Time) × 2 (Group) RANOVA was used for each fitness test.	For men, significant effects of Time were found for flexibility, muscular endurance and strength. These data provide evidence of multiple improvements in fitness after six months of CrossFit participation, with the greatest improvement in 1.5-mile run time among the least experienced women.
Tibana, de Sousa [46]	9 men, average age 27.7 ± 3.2 years, average body fat percentage 11.3 ± 4.6%. With an average experience of 41.1 ± 19.6 months in training, which allowed them to have an adequate level of physical condition for the CrossFit^®^ sessions analysed.	To analyse the physiological responses and perception of effort in short (4 min) and long (17 min) CrossFit sessions, measuring blood lactate, HR and RPE.	Two CrossFit^®^ sessions were performed with measurements of blood lactate, HR and perceived exertion before, during and after exercise. Lactate accumulation and recovery, HR during exercise and post-session RPE were assessed.	Short CrossFit^®^ sessions generated higher lactate levels compared to long sessions, but there were no significant differences in HR or RPE between the two.
Crawford, Drake [47]	25 healthy participants (13 males and 12 females) with mean ages of 22.6 ± 3.5 years for males and 21.0 ± 1.5 years for females.	To determine whether HIFT-induced improvements in physical work capacity are related to changes in aerobic capacity, maximal strength and peak power.	Participants underwent a six-week HIFT intervention, assessing VO_2_max, maximal strength (squats, shoulder press, deadlift), peak power, fatigue index and physical work capacity before and after the intervention.	Following the HIFT intervention, significant improvements were observed in all physiological measures, and the relationships between these and physical work capacity were strengthened. However, physiological changes did not significantly predict changes in physical work capacity.
Claudino, Gabbett [7]	Systematic review and meta-analysis including 31 CrossFit studies. Of these, only two had a high level of evidence and low risk of bias. The majority of included studies showed low levels of evidence and high risk of bias.	To analyse the findings of the scientific literature related to CrossFit through a systematic review and meta-analysis.	PubMed, Web of Science, Scopus, Bireme/MedLine and SciELO were systematically searched for the effects of CrossFit. The review followed PRISMA guidelines and assessed the evidence using the Oxford Levels of Evidence. Only studies on CrossFit as a training programme were included, calculating effect sizes and assessing heterogeneity with a random-effects model.	Most of the studies analysed had low levels of evidence and high risk of bias, suggesting the need to improve methodology in future research. Although no significant results were found in the meta-analysis, preliminary data indicate that CrossFit is associated with higher levels of sense of community, satisfaction and motivation.
Brisebois, Rigby [48]	14 physically inactive adults (4 men and 10 women) with no HIFT experience underwent high-intensity functional training sessions three times a week for eight weeks.	To assess physiological and fitness adaptations in physically inactive adults after eight weeks of high-intensity functional training.	Participants performed HIFT sessions three times a week for eight weeks. Resting HR, blood pressure, VO_2_max, body composition and physical performance (leg press, bench press, one-minute sit-up and seated reach tests) were assessed before and after the intervention.	A significant decrease in resting HR and diastolic blood pressure was observed, while systolic blood pressure did not change. There were improvements in VO_2_max, lean body mass and physical performance (leg press, bench press, one-minute sit-ups, and seated reach). Fat mass did not show significant changes.

HIFT (High-Intensity Functional Training); HICT (High-Intensity Continuous Training); PRISMA (Preferred Reporting Items for Systematic Reviews and Meta-Analyses); VO_2_ (oxygen consumption); BMI (body mass index); HRV (heart rate variability); HR (heart rate); BDNF (brain-derived neurotrophic factor); CK (creatine kinase); RPE (Perception of Exertion); PRS (Perceived Recovery Status); NMES (Neuromuscular Surface Electrical Stimulation); VAMa (Maximal Continuous Aerobic Speed); VAMi (Maximal Intermittent Aerobic Speed); AMRAP (As many reps as possible); RER (Repetitions in reserve); PP (Peak Power); AP (Average Power); FI (Fatigue Index); TW (Total Work); WG (Total Workload).

The synthesis of the results was structured along four main thematic axes:

### 3.1. Physiological Demands

Nine studies [8,14,15,17,21,22,26,27,28] analysed physiological responses revealing that HIFT methods markedly improve VO_2_max, maximal strength, power and localised muscular endurance. These capabilities are essential to tolerate the cumulative stress of consecutive workouts. A combination of high-intensity intermittent efforts with sustained loads, which require high energy efficiency and recovery capacity, was observed. Along these lines, a study simulating a full HYROX competition showed a high cardiovascular demand, with approximately 80% of the time in very high heart rate zones (41.6% in Zone 4 [80–90% HRmax] and 50.5% in Zone 5 [90–100% HRmax]), as well as significant levels of lactate and perceived exertion, especially in the final stations [8]. Studies such as Brandt et al. and Wang et al. [8,14], using meta-analysis, confirmed substantial improvements in VO_2_max, maximum strength, and lactate tolerance. D’Hulst et al. [19] documented advanced physiological profiles in elite athletes with VO_2_ values of up to 4.64 L/min in men.

### 3.2. Predictors of Performance

Eight studies [8,16,17,18,19,24,35,40] identified key variables associated with hybrid competition performance. Key predictors included lean mass, lower body strength, VO_2_ peak, functional sports experience and optimised body composition. Research in elite CrossFit athletes showed that the ability to generate power and sustain it under fatigue is critical in fast transitional formats [19]. Complementarily, it has been verified that higher VO_2_max, lower body fat percentage and high volume of resistance training correlated with better performance [8,40], underlining the importance of aerobic fitness and structural efficiency in this type of disciplines.

Zeitz, Cook [40] found a negative correlation between VO_2_max and completion time in the WOD “Fran” (*r* = −0.72). Similarly, Ponce-García, García-Romero [16] and Mangine, Tankersley [41] that lean mass and competitive experience are robust predictors of performance in high-intensity tests.

### 3.3. Adaptations to Training

Six articles [14,20,31,36,45,47] addressed the adaptations induced by HIFT programmes. Significant improvements in aerobic and anaerobic capacity, neuromuscular parameters and metabolic efficiency were documented. In addition, it was shown that high-intensity sessions generate a physiological environment that stimulates hormonal, autonomic and immunological responses relevant to the improvement of functional performance [21,26,27,28]. Crawford, Drake [47] and Luis Mate-Munoz, Budurin [31] demonstrated increases in strength, anaerobic power, and metabolic efficiency after 6-week interventions. Hollerbach, Cosgrove [36] showed significant improvements in muscle strength and endurance after university HIFT programmes.

### 3.4. Psychobiological Responses

Four studies [15,27,29,30] examined perception of exertion, subjective fatigue and recovery. Prior functional training experience was associated with better fatigue tolerance, greater cognitive efficiency during exertion and less performance decline in prolonged testing. These psychobiological variables are particularly relevant in hybrid competitions, where mental resilience may determine performance consistency.

Santos, Morais [15] observed a greater perception of effort and lactate concentration in HIFT sessions compared to HICT. De Brito, Fernandes [27] found that more experienced athletes showed less cognitive fatigue and more efficient execution times during the “Fran” WOD.

In addition to the thematic categorisation, several studies reported concrete quantitative data on physiological improvements. For instance, studies such as Wang, Soh [14] and D’Hulst, Hodzic [19] reported increases in VO_2_max of between 8 and 15% following HIFT programmes. In terms of maximum strength, improvements of 10 to 20% in 1RM were documented in exercises such as squats or thrusters (Kapsis, Tsoukos [32], Leitao, Dias [35]). Likewise, localised muscle endurance increased by 12 to 25% in functional exercises and maximum repetition tests (Hollerbach, Cosgrove [36], Adami, Rocchi [33]). These improvements are relevant for withstanding the cumulative load of hybrid competitions, where the ability to maintain performance under fatigue is critical.

Although not all studies differentiated by sex, Ponce-García, García-Romero [16] reported that men have higher absolute values of anaerobic power, although the differences are reduced when adjusted for muscle mass. In terms of experience level, Adami, Rocchi [33] and De Brito, Fernandes [27] showed that advanced athletes have higher VO_2_max levels, maximum strength, and better fatigue tolerance. In terms of modality, only Brandt, Ebel [8] specifically analysed the HYROX format, showing a majority cardiovascular load (80% of the time in Z4 and Z5), while most studies focused on CrossFit-type protocols. Therefore, the lack of comparative data prevented a more in-depth analysis between disciplines.

Based on a simplified assessment of methodological quality based on design, assessment tools used, and internal validity criteria, the 39 studies included in this review were classified as follows: 12 studies were considered high quality (systematic reviews, meta-analyses, or robust experimental designs), 17 were of moderate quality (well-structured observational studies), and 10 were of low methodological quality (narrative reviews, small samples, or no statistical control).

In order to facilitate the overall interpretation of the findings, a summary of the four thematic areas identified is presented below, together with the number of associated studies, the main results found, representative studies, and the predominant methodological quality. This table provides an integrated view of the effects of high-intensity functional training (HIFT) in hybrid competition contexts, as well as the areas with the greatest scientific rigour and those where evidence gaps persist (Table 2).

## 4. Discussion

The main finding of this scoping review is that high-intensity functional training (HIFT) consistently enhances key physical attributes such as strength, power, and aerobic capacity in trained populations [14,17]. These improvements are attributed to neuromuscular adaptations, increased metabolic efficiency, and greater training density, all of which are essential in hybrid competitions where repeated strength-endurance efforts are required. Moreover, the reviewed evidence highlights the psychophysiological demands of HIFT, especially in competitive formats like CrossFit and HYROX, where minimal rest intervals amplify cumulative load and fatigue-related responses [8,15].

Despite the observed consistency in key outcomes, methodological limitations across the included studies restrict the strength and generalisability of the conclusions. Many studies employed small sample sizes, e.g., n < 20 [15,17], which increases the risk of statistical noise and reduces external validity. Additionally, considerable heterogeneity was observed in terms of training protocols, session structures, and outcome measures (e.g., different WODs, durations, or instruments). This variability complicates direct comparison and limits the precision with which improvements can be attributed to specific HIFT formats or intensities. While positive trends are clear, caution is needed when extrapolating results to broader or non-athletic populations.

These findings align with the interference effect model, which posits that simultaneous demands on strength and endurance systems can hinder maximal adaptation if not properly sequenced or recovered. The cumulative fatigue and neuromuscular stress described in several studies [8,27] illustrate the need for structured programming that minimises concurrent interference. Likewise, the theory of adaptation pathways is supported by the observation that training outcomes vary depending on intensity, exercise order, and training history—suggesting that chronic exposure to HIFT may channel adaptations more efficiently when stimuli are periodised and strategically targeted.

It is important to acknowledge that not all findings were consistent. For instance, while most studies reported positive correlations between VO_2_max and performance [40], others failed to identify any single physiological predictor with strong explanatory power [25]. Similarly, although strength improvements were widely documented, few studies differentiated results by sex or training level [16], limiting generalisation. Additionally, outcomes related to recovery varied depending on whether passive or active rest was applied, and definitions and protocols differed across studies, reducing comparability. These discrepancies highlight the need for more standardised research designs within the HIFT literature.

Another relevant consideration is the impact of accumulated fatigue on movement technique, which may increase injury risk if not properly managed [16,35]. Consequently, structured training planning, appropriate workload distribution, and tailored recovery strategies are crucial to maximising performance while preserving athlete safety. Evidence also suggests that athletes with greater experience tolerate fatigue more effectively, maintaining technique quality under pressure [27,33]. Furthermore, the nature of rest between efforts influences recovery: passive rest appears to promote physiological restoration, while active rest maintains cardiovascular load—an important consideration in hybrid formats where rest intervals are minimal.

The use of physiological and biomechanical monitoring tools (e.g., heart rate variability, motion analysis) is recommended to adjust training loads and prevent overuse-related issues.

The predominance of aerobic contribution in HYROX, as evidenced by the high proportion of time spent in upper heart rate zones (~80%) and elevated lactate levels [8], reinforces the importance of aerobic efficiency and fatigue resistance in this format. However, the limited number of studies specifically addressing HYROX restricts the ability to draw firm, modality-specific conclusions, especially compared to the more extensive body of research on CrossFit. Differences in repetition volume, rest structure, and exercise specificity likely modulate both acute responses and chronic adaptations.

Future research should prioritise well-designed longitudinal studies with standardised HIFT protocols to better understand long-term adaptations. Specifically, randomised controlled trials comparing CrossFit and HYROX formats over 8–12 weeks would allow clearer insights into modality-specific effects. Key outcomes should include VO_2_max, lactate threshold, neuromuscular fatigue, hormonal markers, and perceived recovery. Samples should be stratified by sex, age, and training level to explore differential adaptations. Furthermore, studies should incorporate both physiological (e.g., HRV, creatine kinase) and perceptual (e.g., RPE, PRS) recovery indicators. Finally, field-based research monitoring injury incidence and biomechanical changes during real competitions would support applied training design and injury prevention strategies in hybrid sport contexts.

## 5. Conclusions

The systematic review indicates that high-intensity functional training generates positive adaptations in strength and endurance, but can also induce significant fatigue, affecting performance and execution technique. In hybrid competitions efficient fatigue and recovery management are key factors to optimise performance and to minimise the risk of injury.

Continuous monitoring of physiological and biomechanical parameters will allow optimisation of training programmes and reduce the risk of excessive fatigue and injury. In addition, future studies should focus on assessing muscle fatigue along with metabolic and physiological analyses for a deeper understanding of the impact of high-intensity training on endurance athletes.

## Figures and Tables

**Table 2:** Summary of thematic axes, number of studies, main findings, key studies, and prevailing methodological quality.

Thematic Axis	No. of Studies	Main Findings	Key Studies	Prevailing Quality
Physiological demands	9	Improvements in VO_2_max (8–15%), maximal strength (10–20%), and lactate tolerance	Wang, Soh [14], Brandt, Ebel [8], Tibana, Dominski [17]	High
Performance predictors	8	VO_2_max, lean mass, lower-body strength, and experience identified as key predictors	Ponce-García, García-Romero [16], D’Hulst, Hodzic [19], Zeitz, Cook [40]	Moderate to high
Training adaptations	6	Enhancements in metabolic efficiency, muscular strength, and anaerobic power	Luis Mate-Munoz, Budurin [31], Hollerbach, Cosgrove [36], Crawford, Drake [47]	Moderate
Psychobiological responses	4	Elevated RPE, variable fatigue tolerance, improved cognitive efficiency with prior experience	Santos, Morais [15], De Brito, Fernandes [27]	Moderate to low

## Data Availability

Data is contained within the article.

## References

[1] Coffey V.G., Hawley J.A. (2017). Concurrent exercise training: Do opposites distract?. J. Physiol..

[2] Schlegel P. (2020). CrossFit^®^ Training Strategies from the Perspective of Concurrent Training: A Systematic Review. J. Sports Sci. Med..

[3] Gao J., Yu L. (2023). Effects of concurrent training sequence on VO(2max) and lower limb strength performance: A systematic review and meta-analysis. Front. Physiol..

[4] Wilson J.M., Marin P.J., Rhea M.R., Wilson S.M., Loenneke J.P., Anderson J.C. (2012). Concurrent training: A meta-analysis examining interference of aerobic and resistance exercises. J. Strength Cond. Res..

[5] Brito M.A., Fernandes J.R., De Carvalho P.H.B., Brito C.J., Aedo-Muñoz E., Soto D.A.S., Miarka B. (2023). Acute Effect of a Cross-Training Benchmark on Psychophysiological Factors of Cross-Training According to Performance. Sport Mont..

[6] Fernando W., Santos W., Barbieri J., de Medeiros Lima L.E., Miguel H., Guedes D., da Silva R.P., Marchioni E., Moriggi R. (2021). Physical capacities and anthropometric measures between Crossfit^®^ practitioners and Crosstraining. Multidiscip. Sci. J..

[7] Claudino J.G., Gabbett T.J., Bourgeois F., Souza H.d.S., Miranda R.C., Mezêncio B., Soncin R., Filho C.A.C., Bottaro M., Hernandez A.J. (2018). CrossFit Overview: Systematic Review and Meta-analysis. Sports Med.-Open.

[8] Brandt T., Ebel C., Lebahn C., Schmidt A. (2025). Acute physiological responses and performance determinants in Hyrox(©)—A new running-focused high intensity functional fitness trend. Front. Physiol..

[9] Booth A., Papaioannou D., Sutton A. (2012). Systematic Approaches to a Successful Literature Review.

[10] Tricco A.C., Lillie E., Zarin W., O’Brien K.K., Colquhoun H., Levac D., Moher D., Peters M.D.J., Horsley T., Weeks L. (2018). PRISMA Extension for Scoping Reviews (PRISMA-ScR): Checklist and Explanation. Ann. Intern. Med..

[11] Arksey H., O’Malley L. (2005). Scoping studies: Towards a methodological framework. Int. J. Soc. Res. Methodol..

[12] Codina L. Scoping Reviews: Características, Frameworks Principales y Uso en Trabajos Académicos, 2022. https://www.lluiscodina.com/scoping-reviews-guia/.

[13] Downes M.J., Brennan M.L., Williams H.C., Dean R.S. (2016). Development of a critical appraisal tool to assess the quality of cross-sectional studies (AXIS). BMJ Open.

[14] Wang X., Soh K.G., Zhang L., Liu X., Ma S., Zhao Y., Sun C. (2025). Effects of high-intensity functional training on physical fitness in healthy individuals: A systematic review with meta-analysis. BMC Public Health.

[15] Santos D.A., Morais N.S., Viana R.B., Costa G.C., Andrade M.S., Vancini R.L., Weiss K., Knechtle B., de Lira C.A. (2025). Comparison of physiological and psychobiological acute responses between high intensity functional training and high intensity continuous training. Sports Med. Health Sci..

[16] Ponce-García T., García-Romero J., Carrasco-Fernández L., Castillo-Domínguez A., Benítez-Porres J. (2025). Sex differences in anaerobic performance in CrossFit^®^ athletes: A comparison of three different all-out tests. PeerJ.

[17] Tibana R.A., Dominski F.H., Andrade A., De Sousa N.M.F., Voltarelli F.A., de Sousa Neto I.V. (2024). Exploring the relationship between Total Athleticism score and CrossFit^®^ Open Performance in amateur athletes: Single measure involving body fat percentage, aerobic capacity, muscle power and local muscle endurance. Eur. J. Transl. Myol..

[18] Martinho D.V., Rebelo A., Gouveia É.R., Field A., Costa R., Ribeiro A.S., Casonatto J., Amorim C., Sarmento H. (2024). The physical demands and physiological responses to CrossFit^®^: A scoping review with evidence gap map and meta-correlation. BMC Sports Sci. Med. Rehabil..

[19] D’hUlst G., Hodžić D., Leuenberger R., Arnet J., Westerhuis E., Roth R., Schmidt-Trucksäss A., Knaier R., Wagner J. (2024). Physiological Profiles of Male and Female CrossFit Athletes. Int. J. Sports Physiol. Perform..

[20] Wang X., Soh K.G., Samsudin S., Deng N., Liu X., Zhao Y., Akbar S., Gardasevic J. (2023). Effects of high-intensity functional training on physical fitness and sport-specific performance among the athletes: A systematic review with meta-analysis. PLoS ONE.

[21] Sant’Ana L.D.O., Evmenenko A., Vianna J.M., Machado S., Teixeira D.S. (2023). Autonomic responses and internal load analysis through acute assessment of heart rate variability after a high-intensity functional training session. Arch. De Med. Del Deporte.

[22] Pearson R.C., Olenick A.A., Jenkins N.T. (2023). Metabolic Response During High-Intensity Interval Exercise and Resting Vascular and Mitochondrial Function In Crossfit Participants. Kinesiology.

[23] Moscatelli F., Messina G., Polito R., Porro C., Monda V., Monda M., Scarinci A., Dipace A., Cibelli G., Messina A. (2023). Aerobic and Anaerobic Effect of CrossFit Training: A Narrative Review. Sport Mont..

[24] Meier N., Sietmann D., Schmidt A. (2023). Comparison of Cardiovascular Parameters and Internal Training Load of Different 1-h Training Sessions in Non-elite CrossFit^®^ Athletes. J. Sci. Sport Exerc..

[25] Meier N., Schlie J., Schmidt A. (2023). CrossFit^®^: ‘Unknowable’ or Predictable?—A Systematic Review on Predictors of CrossFit^®^ Performance. Sports.

[26] McDougle J.M., Mangine G.T., Townsend J.R., Jajtner A.R., Feito Y. (2023). Acute physiological outcomes of high-intensity functional training: A scoping review. PeerJ.

[27] Brito M.A., Fernandes J.R., Carvalho P.H.B.D., Brito C.J., Muñoz E.A., Soto D., Miarka A.S.B. (2023). Acute effect of high-intensity functional training (HIFT) using a benchmark on cognition and physiological parameters according to the competitive level. J. Phys. Educ. Sport.

[28] Sousa Neto I.V.D., Sousa N.M.F.D., Neto F.R., Falk Neto J.H., Tibana R.A. (2022). Time-course effects of functional fitness sessions performed at different intensities on the metabolic, hormonal, and BDNF responses in trained men. BMC Sports Sci. Med. Rehabil..

[29] Neto I.V.d.S., de Sousa N.M.F., Neto F.R., Neto J.H.F., Tibana R.A. (2022). Time Course of Recovery Following CrossFit^®^ Karen Benchmark Workout in Trained Men. Front. Physiol..

[30] Martinez-Gomez R., Valenzuela P.L., Lucia A., Barranco-Gil D. (2022). Comparison of Different Recovery Strategies After High-Intensity Functional Training: A Crossover Randomized Controlled Trial. Front. Physiol..

[31] Maté-Muñoz J.L., Budurin M., González-Lozano S., Heredia-Elvar J.R., Cañuelo-Márquez A.M., Barba-Ruiz M., Muriarte D., Garnacho-Castaño M.V., Hernández-Lougedo J., García-Fernández P. (2022). Physiological Responses at 15 Minutes of Recovery after a Session of Functional Fitness Training in Well-Trained Athletes. Int. J. Environ. Res. Public Health.

[32] Kapsis D.P., Tsoukos A., Psarraki M.P., Douda H.T., Smilios I., Bogdanis G.C. (2022). Changes in Body Composition and Strength after 12 Weeks of High-Intensity Functional Training with Two Different Loads in Physically Active Men and Women: A Randomized Controlled Study. Sports.

[33] Adami P.E., Rocchi J.E., Melke N., Macaluso A. (2022). Physiological comparison between competitive and beginner high intensity functional training athletes. J. Hum. Sport Exerc..

[34] Adami P.E., Rocchi J.E., Melke N., De Vito G., Bernardi M., Macaluso A. (2022). Physiological profile comparison between high intensity functional training, endurance and power athletes. Eur. J. Appl. Physiol..

[35] Leitão L., Dias M., Campos Y., Vieira J.G., Sant’Ana L., Telles L.G., Tavares C., Mazini M., Novaes J., Vianna J. (2021). Physical and Physiological Predictors of FRAN CrossFit(R) WOD Athlete’s Performance. Int. J. Environ. Res. Public Health.

[36] Hollerbach B.S., Cosgrove S.J., DeBlauw J.A., Jitnarin N., Poston W.S.C., Heinrich K.M. (2021). Muscular Strength, Power, and Endurance Adaptations after Two Different University Fitness Classes. Sports.

[37] Bustos-Viviescas B.J., Acevedo-Mindiola A.A., Osorio R.D.M. (2021). Relationship of continuous and intermittent maximal aerobic speed to crossfit® wod karen performance in physically active subjects. Rev. Cuba. De Investig. Biomed..

[38] Ben-Zeev T., Okun E. (2021). High-Intensity Functional Training: Molecular Mechanisms and Benefits. Neuromolecular Med..

[39] Adami P.E., Rocchi J.E., Melke N., Macaluso A. (2021). Physiological profile of high intensity functional training athletes. J. Hum. Sport Exerc..

[40] Zeitz E.K., Cook L.F., Dexheimer J.D., Lemez S., Leyva W.D., Terbio I.Y., Tran J.R., Jo E. (2020). The Relationship between CrossFit^®^ Performance and Laboratory-Based Measurements of Fitness. Sports.

[41] Mangine G.T., Tankersley J.E., McDougle J.M., Velazquez N., Roberts M.D., Esmat T.A., VanDusseldorp T.A., Feito Y. (2020). Predictors of CrossFit Open Performance. Sports.

[42] Cavedon V., Milanese C., Marchi A., Zancanaro C. (2020). Different amount of training affects body composition and performance in High-Intensity Functional Training participants. PLoS ONE.

[43] Neto J.H.F., Kennedy M.D. (2019). The Multimodal Nature of High-Intensity Functional Training: Potential Applications to Improve Sport Performance. Sports.

[44] Feito Y., Giardina M.J., Butcher S., Mangine G.T. (2019). Repeated anaerobic tests predict performance among a group of advanced CrossFit-trained athletes. Appl. Physiol. Nutr. Metab..

[45] Cosgrove S.J., Crawford D.A., Heinrich K.M. (2019). Multiple Fitness Improvements Found after 6-Months of High Intensity Functional Training. Sports.

[46] Tibana R.A., de Sousa N.M.F., Prestes J., Voltarelli F.A. (2018). Lactate, heart rate and rating of perceived exertion responses to shorter and longer duration crossfit^®^ training sessions. J. Funct. Morphol. Kinesiol..

[47] Crawford D.A., Drake N.B., Carper M.J., DeBlauw J., Heinrich K.M. (2018). Are Changes in Physical Work Capacity Induced by High-Intensity Functional Training Related to Changes in Associated Physiologic Measures?. Sports.

[48] Brisebois M.E., Rigby B.R., Nichols D.L. (2018). Physiological and Fitness Adaptations after Eight Weeks of High-Intensity Functional Training in Physically Inactive Adults. Sports.

